# Impaired Kv7 channel function in cerebral arteries of a tauopathy mouse model (rTg4510)

**DOI:** 10.14814/phy2.13920

**Published:** 2018-12-11

**Authors:** Inge E. M. de Jong, Thomas A. Jepps

**Affiliations:** ^1^ Department of Neurodegeneration Lundbeck A/S Valby Denmark; ^2^ Department of Biomedical Sciences Faculty of Health and Medical Sciences University of Copenhagen Copenhagen Denmark

**Keywords:** cerebral artery, KCNE, KCNQ, Kv7 Channels, rTg4510, tauopathies

## Abstract

In tauopathies, such as Alzheimer's disease with or without concomitant amyloid *β* plaques, cerebral arteries display pathological remodeling, leading to reduced brain tissue oxygenation and cognitive impairment. The precise mechanisms that underlie this vascular dysfunction remain unclear. Kv7 voltage‐dependent K^+^ channels contribute to the development of myogenic tone in rat cerebral arteries. Thus, we hypothesized that Kv7 channel function would be impaired in the cerebral arteries of a tauopathy mouse model (rTg4510), which might underlie cerebral hypoperfusion associated with the development of neurofibrillary tangles in tauopathies. To test our hypothesis we performed wire myography and quantitative PCR on cerebral arteries, mesenteric arteries and the inferior frontotemporal region of the brain surrounding the middle cerebral artery from tau transgenic mice (rTg4510) and aged‐matched controls. We also performed whole‐cell patch clamp experiments on HEK293 cells stably expressing Kv7.4. Here, we show that Kv7 channels are functionally impaired in the cerebral arteries of rTg4510 mice, but not in mesenteric arteries from the same mice. The quantitative PCR analysis of the cerebral arteries found no change in the expression of the genes encoding the Kv7 channel *α*‐subunits, however, we found reduced expression of the ancillary subunit, KCNE5 (also termed KCNE1L), in the cerebral arteries of rTg4510 mice. In the brain, rTg4510 mice showed reduced expression of Kv7.3, Kv7.5, and Kv2.1. Co‐expression of KCNE5 with Kv7.4 in HEK293 cells produced larger currents at voltages >0 mV and increased the deactivation time for the Kv7.4 channel. Thus, our results demonstrate that Kv7 channel function is attenuated in the cerebral arteries of Tg4510 mice, which may result from decreased KCNE5 expression. Reduced Kv7 channel function might contribute to cerebral hypoperfusion in tauopathies, such as Alzheimer's disease.

## Introduction

The prevalence of neurodegenerative diseases is increasing worldwide with dementias being responsible for the greatest burden of the disease (Prince et al. 2013). One group of neurodegenerative diseases, which result from the gradual and progressive loss of neural cells, are characterized by the formation of neurofibrillary tangles (NFTs) arising from dysfunction and aggregation of the microtubule‐associated protein tau. These conditions are known as tauopathies and include Alzheimer's disease (AD), Pick's disease and frontotemporal dementia with parkinsonism linked to chromosome 17 (FTDP‐17). AD remains the most common cause of dementia and NFTs have been detected in the early neurodegeneration stages before fully developed AD, regardless of concomitant amyloid *β* plaques (Braak and Braak 1991; Duyckaerts et al. 2015). It is now known that AD is characterized by cerebrovascular remodeling, which occurs before the onset of neurodegeneration, increasing vascular resistance in the cerebral circulation and decreasing cerebral blood flow (Zhao et al. 2014; Bradley et al. 2002; Merlini et al. 2016; Qiu et al. 2016). In particular, cerebral arteries in tauopathies display early pathological vessel wall remodeling, leading to reduced brain tissue oxygenation and cognitive impairment (Perry et al. 1998; Vidal et al. 2000; Stopa et al. 2008; Merlini et al. 2016). The precise mechanisms that underlie this vascular dysfunction remain unclear.

Kv7 channels are voltage‐dependent K^+^ channels, encoded for by the KCNQ genes, which are important determinants of the resting membrane potential in vascular and non‐vascular smooth muscle cells (Jepps et al. 2013; Greenwood and Tribe 2014; Stott et al. 2014; Fosmo and Skraastad 2017; Byron and Brueggemann 2018), the action potential propagation in neurons (Wang and Li 2016), and the major repolarization current in the heart (I_Ks_) (Barhanin et al. 1996; Sanguinetti et al. 1996). In smooth muscle cells of rat cerebral arteries, Kv7.4 and Kv7.5 channels regulate myogenic tone, with inhibition of these channels resulting in increased cerebral artery resistance at physiological pressures (Zhong et al. 2010a; Mani et al. 2011, 2013).

Given the importance of Kv7 channels in regulating cerebral artery tone and the decreased cerebral blood flow associated with tauopathies related to AD, we hypothesized that Kv7 channel function would be impaired in the cerebral arteries of a tauopathy mouse model (rTg4510), which might underlie cerebral hypoperfusion associated with the development of NFTs and AD. The rTg4510 mouse model of tauopathy carries human tau containing the P301L mutation (4R0N) linked to familial frontotemporal dementia (described in Ramsden et al. (2005)) and displays age‐dependent learning and memory impairments, hyperactivity and neurodegeneration correlating with tau pathology (Santacruz et al. 2005; Ramsden et al. 2005; Cook et al. 2014; Jul et al. 2016; Helboe et al. 2017). Thus, the aim of this study was to investigate the function and expression of Kv7 channels (and associated proteins) in cerebral arteries of the rTg4510 mouse model of tau hyperphosphorylation and age‐matched control mice.

## Material and Methods

### Animals

Male wild‐type (WT) and the rTg(tau_P301L_)4510 mice (11 months old) were used in this study. The rTg4510 strain is created by crossing two transgenic parental strains. One contains P301L‐hTau downstream of an inducible tetracycline‐operonresponder (TRE) promoter. The second contains a tetracycline‐responsive transcriptional activator (tTA) driven by the CaMKII*α* promoter. The tTA ensures doxycycline‐dependent expression, with a 13‐fold higher hTau protein expression than the endogenous mouse tau protein levels in the absence of doxycycline (Santacruz et al. 2005; Ramsden et al. 2005). Mice in this study were not administered doxycycline. The CaMKII*α* promoter drives expression of the P301L‐hTau transgene primarily in forebrain (including hippocampal and cortical) neurons. The F1 progeny of the two transgenic parental strains (rTg4510) carries responder and activator transgenes, necessary for the expression of the tau transgene. Mice expressing the tTA activator transgenes were maintained on 129S6 background strain (Taconic) and mutant tau responder mice were maintained in the FVB/NCrl background strain (Taconic). The wildtype, non‐Tg littermates were of the FBV/129 background. Mice were screened by PCR using the following primer pairs 5′‐GATTAACAGCGCATTAGAGCTG‐3′ and 5′‐GCATATGATCAATTCAAGGCCGATAAG‐3′ for the tTA activator transgene and 5′‐TGAACCAGGATGGCTGAGCC‐3′ and 5′‐TTGTCATCGCTTC CAGTCCCCG‐3′ for the mutant tau responder transgene. rTg4510 and non‐Tg littermate F1 mice were bred at Taconic, Denmark. The mice were group‐housed (5 animals/cage) and received water and food ad libitum (Brogaarden, Denmark) as well as environmental enrichment. The light/dark cycle was 12 h; room temperature was 21 ± 2°C and a relative humidity of 55 ± 5%. Following termination by cervical dislocation, the mesenteric vascular bed and brain were excised and placed in cold physiological salt solution (PSS; composition in mM: NaCl 121; KCl 2.82; KH_2_PO_4_ 1.18; MgSO_4_.7H_2_O 1.17; NaHCO3 25; CaCl_2_ 1.6; EDTA 0.03; glucose 5.5) saturated with carbogen (O_2_ 95%; CO_2_ 5%) at pH 7.4. This study was approved by the National Ethics Committee, Denmark, and performed in accordance with Directive 2010/63/EU on the Protection of Animals Used for Scientific Purposes (European Commission, 2010).

### Myography

Segments (1–2 mm length) of third‐order mesenteric arteries and middle cerebral arteries were isolated from the WT and rTg4510 mice, mounted on 25 *μ*m steel wires and placed under isometric tension in chambers of a wire myograph system (model 620M; Danish Myo Tehcnology (DMT), Aarhus, Denmark) containing PSS maintained at 37°C and saturated with O_2_ 95%; CO_2_ 5%. Each artery segment was normalized by a passive stretch‐tension protocol, as previously described (Mulvany and Halpern 1977). WT arteries had a mean diameter of 160.7 ± 12.96 *μ*m compared to 141 ± 8.30 *μ*m in rTg4510 mice (*n* = 9; *P *=* *0.2174 according to an unpaired Students *t* test). In all arteries, the endothelium was removed, which was tested by application of acetylcholine (data not shown). Cerebral arteries were constricted with either the *α*_1_‐adrenoceptor agonist, methoxamine (10 *μ*mol/L), or the thromboxane A_2_ agonist, U46619 (0.1 *μ*mol/L); and the mesenteric arteries were constricted with methoxamine (10 *μ*mol/L). Once a stable contraction had been generated, the Kv7.2‐7.5 channel activator, S‐1, was applied at 3 and 10 *μ*mol/L. Changes in force (mN) were recorded with a Powerlab 8/25‐LabChart 7 Pro data acquisition system (AD Instruments Ltd., Oxford, United Kingdom).

### Quantitative polymerase chain reaction (qPCR)

qPCR analysis of KCNQ1‐5, KCNE3‐5, and KCNB1 in the cerebral arteries, mesenteric arteries, and the neuronal tissue of the inferior frontotemporal region surrounding the middle cerebral artery of WT and Tg4510 mice was performed as described previously in Jepps et al. (2014). Briefly, RNA was extracted using the RNeasy Micro Extraction Kit (Qiagen) and reverse transcribed using Precision nanoScript2 Reverse Transcription Kit (Primerdesign Ltd. Southampton, U.K.). Duplicate reactions were performed in 20 *μ*L volumes containing 10 *μ*L Precision‐iC SYBR green master mix (PrimerDesign Ltd.), 10 ng cDNA, 300 nmol/L primer (PrimerDesign Ltd.) and made up to 20 *μ*L with nuclease‐free water. The following cycling conditions were used in the CFX96 Real‐Time PCR Detection System (Bio‐Rad, Denmark): initial activation at 95°C for 10 min, followed by 40 cycles of 95°C for 15 sec, and 60°C for 1 min, and data were collected during each cycling phase. Melt curve analysis was performed to ensure each primer set amplified a single, specific product. RT‐ samples and no‐template controls (NTCs) were run to assess contamination. Quantification cycle (Cq) values were determined using Bio‐Rad CFX96 Manager 3.0 software and the single threshold mode. The expression levels of the genes of interest are expressed relative to the mean expression levels of the reference genes (Livak and Schmittgen 2001).

The mouse geNorm reference gene selection kit (Primerdesign Ltd.) was used to identify the most stable reference genes and to determine optimal number of reference genes required for reliable normalization of the genes of interest in our samples (Vandesompele et al. 2002). The reference genes used for cerebral and mesenteric arteries were *β*‐actin and cytochrome C1. For the brain, the most stable reference genes were *β*‐actin and Tyrosine 3‐Monooxygenase/Tryptophan 5‐Monooxygenase Activation Protein Zeta (YWHAZ). All primers (Table 1) were designed and optimized by Primerdesign Ltd. The sequences for the reference genes are commercially sensitive and therefore unavailable from Primerdesign Ltd.

**Table 1:** Primers used in this study, designed and optimized by Primerdesign Ltd

Gene	Accession number	Product length	Sequence
Kcnq1	NM_008434	75	5′‐CTCGGAGTCACACGCTTCT‐3′
			3′‐GCTTGAACTTCTTCTTCTTTACCAT‐5′
Kcnq2	NM_010611	111	5′‐CCCTCATTGGTGTCTCGTTCT‐3′
			3′‐GGTTCCGCCGTTTCTCAAAG‐5′
Kcnq3	NM_152923	93	5′‐GAAGAGGGGCAGAGGAGGA‐3′
			3′‐CCTGTACTTGGCGTTGTTCC‐5′
Kcnq4	NM_001081142	112	5′‐GTGGTCTTTGGCTTGGAGTATAT‐3′
			3′‐CGATGACACAGAAGGGTTTCC‐5′
Kcnq5	NM_001160139	107	5′‐GTCAGATAAGAAGAGCCGAGAGA‐3′
			3′‐CGATGGACTGGACCTGTTTCT‐5′
Kcne1	NM_008424	111	5′‐GTTTCCCCAAATCTCTCCACATT‐3′
			3′‐AGCACACACTTCCCATTTCAAA‐5′
Kcne2	NM_134110	97	5′‐CCTGGTATTTAACTGAGTTGGACAT‐3′
			3′‐GCACTGGGGGCTCTTGAAT‐5′
Kcne3	NM_001190869	99	5′‐CTCAACCATATCAAGCCACAGT‐3′
			3′‐GCCTATCAGTCCCTCTTCTCT‐5′
Kcne4	NM_021342	88	5′‐GGAGGAGGGGGCTGATGA‐3′
			3′‐CTGGTGGATGTTCTCGGAAGA‐5′
Kcne5	NM_021487	146	5′‐GCACGAAGAGACCTCAGACAT‐3′
			3′‐GGACAGGAAAACAAGAACACCAT‐5′
Kcnb1	NM_008420	77	5′‐CCAGTCTCAACCCATCCTCAA‐3′
			3′‐TGCTGCCCATCTCCAGTTC‐5′

### HEK cell electrophysiology

Monoclonal HEK293 cells stably expressing human Kv7.4 (Søgaard et al. 2001) were grown in Dulbecco's modified Eagle's medium (Substrate Department, Panum Institute, Copenhagen, Denmark), supplemented with 10% fetal bovine serum (Th Geyer, Denmark), Glutamax (Substrate Department, Panum Institute, Copenhagen, Denmark), 1% penicillin streptomycin (Sigma) and incubated at 37°C in 5% CO_2_. Cells were transfected with human KCNE5 using siLentFectTM Lipid (Bio‐Rad, Copenhagen, Denmark), according to manufacturer's instructions. K^+^ currents were recorded from these cells using an Axopatch 700B patch clamp amplifier (Molecular Devices, Sunnydale, CA) generated and digitized using a Digidata 1322A hosted by a PC running pClamp 10.4 software (Molecular Devices, Sunnydale, CA). Membrane current recordings were made using *β*‐escin (50 *μ*mol/L, Sigma, MO) perforated‐patch technique in voltage‐clamp mode. Fire‐polished patch pipettes had a resistance of 3–6 MΩ when filled with a pipette solution of (mM): K^+^ Gluconate (110), KCl (30), MgCl_2_ (0.5) HEPES (5), and EGTA (0.1), pH 7.3. The external solution contained (mM): NaCl (145), KCl (4), MgCl_2_ (1), CaCl_2_ (2), Hepes (10), and glucose (10), pH 7.4. Control I/V's were determined from a holding potential of −80 mV using a step protocol with +10 mV steps to a maximum potential of 50 mV, with 750 msec duration, returning to −30 mV to measure the tail current. For each test potential the maximum current amplitude was taken.

## Results

### Myography

**Figure 1:**
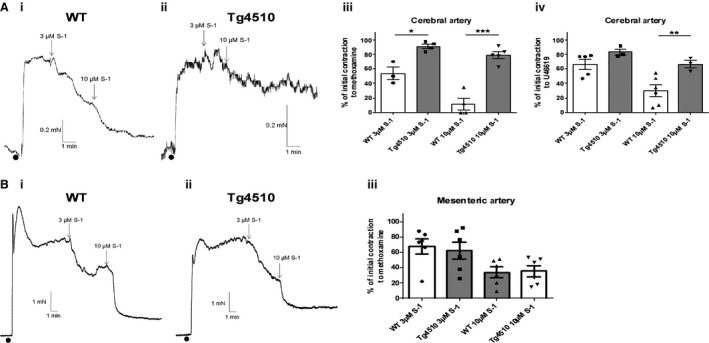
Responses to the Kv7 channel activator, S‐1, are impaired in cerebral arteries of Tg4510 mice compared to WT mice. Representative traces of (Ai) WT and (Aii) rTg4510 mouse cerebral arteries contracted with U46619 before application of S‐1. Mean data showing the relaxations of the cerebral arteries to S‐1 after preconstriction with either (Aiii) methoxamine or (Aiv) U46619 (*n* = 4). (B) (Bi and Bii) Representative traces of the same experiments performed on the mesenteric arteries of WT and rTg4510 mice preconstricted with methoxamine, followed by (Biii) the mean data (*n* = 6). The vasorelaxations to S‐1 were the same in mesenteric arteries from WT and Tg4510 mice. *, ** and *** denote significance of *P* < 0.05, *P* < 0.01, and *P* < 0.001, respectively, according to an unpaired t test with Welch's correction.

In WT mouse middle cerebral arteries, the Kv7.2‐7.5 activator, S‐1 (3 and 10 *μ*mol/L), produced concentration‐dependent relaxations in the presence of both methoxamine and U46619 (*n* = 4; Fig. 1A). The effect of S‐1 was attenuated in middle cerebral arteries from rTg4510 mice at 3 and 10 *μ*mol/L in the presence of methoxamine and at 10 *μ*mol/L in the presence of U46619 (*n* = 4; Fig. 1A). Both WT and rTg4510 middle cerebral arteries relaxed 100 ± 0% to 1 *μ*mol/L nicardipine, the voltage‐gated Ca^2+^ channel blocker (data not shown; *n* = 3 for each). To determine whether Kv7 channel dysfunction in the rTg4510 mice occurred in other arteries, we performed isometric tension measurements on mesenteric arteries from the same mice. In contrast to the cerebral arteries, S‐1 was equally effective at relaxing mesenteric artery segments from WT and rTg4510 mice (*n* = 6; Fig. 1B).

### qPCR

**Figure 2:**
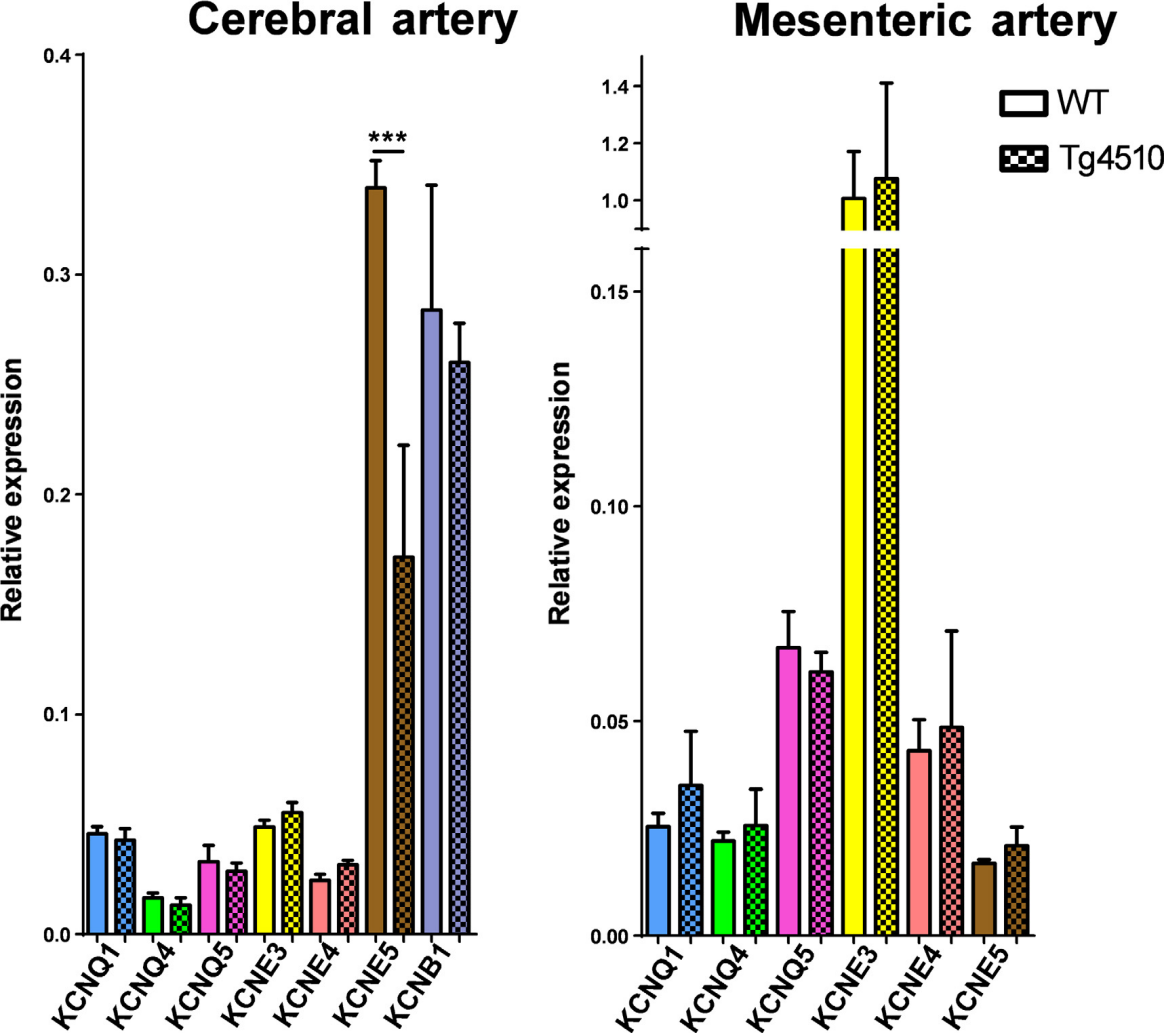
qPCR analysis of specific KCNQ and KCNE isoforms in cerebral arteries and mesenteric arteries of WT and Tg4510 mice (*n* = 3 for each point). Differences in individual gene expression between WT and Tg4510 mice were compared using a one‐way ANOVA followed by a Bonferroni multiple comparisons test (* and *** denote significance of *P* < 0.05 and *P* < 0.001, respectively).

We investigated whether the KCNQ or KCNE mRNA expression was different in the middle cerebral arteries between WT and rTg4510 mice. Expression of the most common vascular KCNQ isoforms, namely KCNE1, KCNQ4 and KCNQ5, were not different between in the middle cerebral arteries in WT and rTg4510 (*N* = 3, with each sample containing middle cerebral arteries pooled from three animals; Fig. 2A). The expression of ancillary subunits KCNE3 and KCNE4 were the same in middle cerebral arteries from WT and rTg4510 mice (Fig. 2A). KCNE5 expression predominated in the middle cerebral arteries of WT mice, which was downregulated in rTg4510 mice (Fig. 2A). We also investigated expression of KCNB1 but there was no difference in the cerebral arteries between WT and rTg4510 mice (Fig. 2A). In mesenteric arteries, KCNE3 expression predominated in both WT and rTg4510 mice; however, in contrast to the middle cerebral artery, none of the genes investigated showed any differences in expression between WT and rTg4510 mice (*N* = 3, with each sample containing mesenteric arteries pooled from three animals).

**Figure 3:**
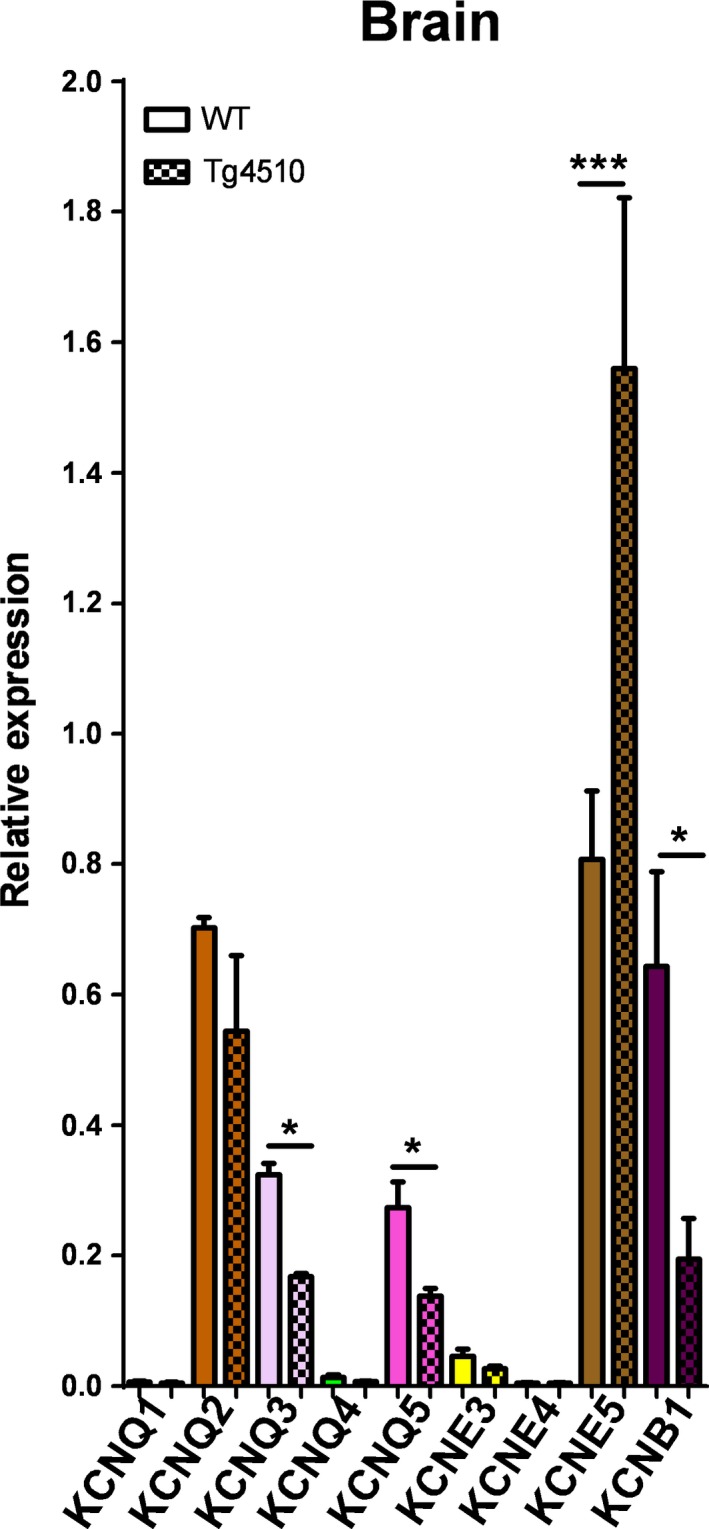
qPCR analysis of specific KCNQ and KCNE isoforms in the brains of WT and Tg4510 mice (*n* = 3 for each point). Differences in individual gene expression between WT and Tg4510 mice were compared using compared using a one‐way ANOVA followed by a Bonferroni multiple comparisons test (* and *** denote *P* < 0.05 and *P* < 0.001, respectively).

We also investigated the expression of all KCNQ isoforms, KCNE3‐5 and KCNB1 in the neuronal tissue of the inferior frontotemporal region surrounding the middle cerebral artery of WT and rTg4510 mice. In this area of the brain, the expression of KCNQ3 and KCNQ5 was downregulated in rTg4510 mice, with the other KCNQ genes unaffected (*n* = 3; Fig. 3). There was no difference in expression of KCNE3 and KCNE4 between WT and rTg4510 mice, but KCNE5 was upregulated in the rTg4510 brain (*n* = 3; Fig. 3). KCNB1 was downregulated in the brains of rTg4510 mice compared to WT (*n* = 3; Fig. 3).

### Electrophysiology

**Figure 4:**
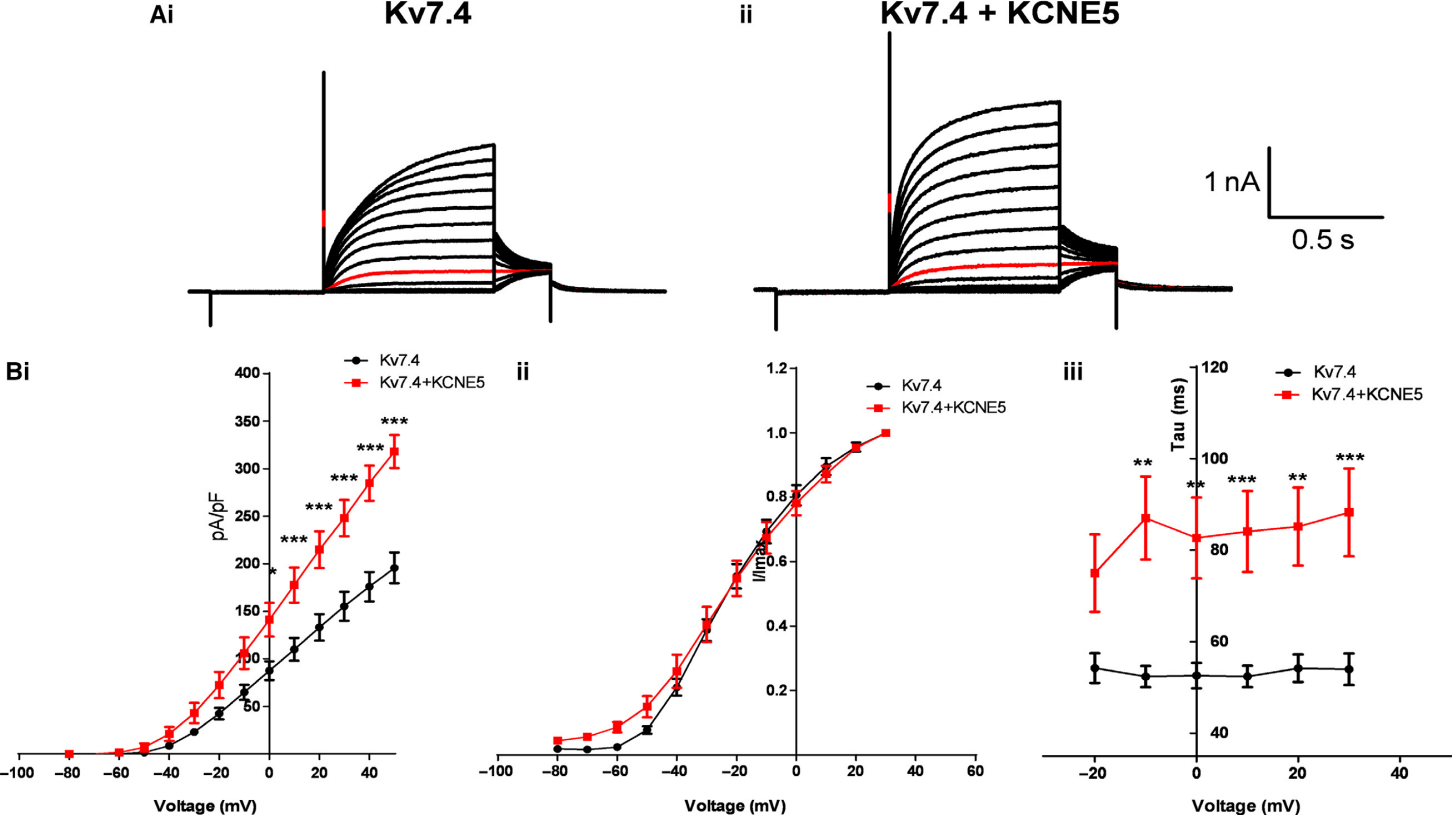
(A) Kv7.4 currents from HEK cells with (*n* = 4) and without (*n* = 5) KCNE5 co‐expressed. (Bi) KCNE5 increased the Kv7.4 current amplitude, (Bii) had no effect on the voltage‐dependence of activation and (Biii) increased deactivation time. *, ** and *** denote significance of *P* < 0.05, *P* < 0.01 and *P* < 0.001, respectively, according to a two‐way ANOVA followed by a Bonferroni post‐test.

We investigated the effect of overexpressed KCNE5 on the Kv7.4 current in HEK cells (Fig. 4; *n* = 4–5). KCNE5 increased the amplitude of the Kv7.4 current at potentials ranging from 0 mV to +50 mV, without affecting the voltage‐dependence of activation (Fig. 4). KCNE5 also increased the Kv7.4 time for deactivation (Fig. 4). The activation kinetics of Kv7.4 were not significantly different in the presence of KCNE5 (data not shown).

## Discussion

Although it is known that cerebrovascular remodeling occurs early in the onset of tauopathies, the precise mechanisms affecting cerebral artery tone in tauopathies remain unknown. As such, the aim of this study was to characterize the function and expression of the Kv7 channels in the cerebral arteries of a tauopathy‐associated dementia mouse model, namely rTg4510, compared to age‐matched control mice. This study is the first to identify a cerebral artery‐specific impairment of Kv7 channel function in a tauopathy‐associated dementia mouse model and we suggest this functional attenuation might be due to a downregulation of the ancillary subunit, KCNE5 in the cerebral arteries of the rTg4510 mice. This is one of the first studies to investigate specific ion channel changes regulating cerebral artery contractility in an animal model of neurodegenerative disease associated with tau hyperphosphorylation.

The Kv7 channels are voltage‐gated potassium channels encoded for by the KCNQ genes (Robbins 2001). There are five isoforms in the Kv7 channel family (Kv7.1‐Kv7.5), and Kv7.4 and Kv7.5 have been shown previously to contribute to the regulation of myogenic tone in rat cerebral and basilar arteries (Zhong et al. 2010a; Mani et al. 2011). Activation of Kv7 channels in pressurized cerebral arteries inhibited myogenic constriction at >20 mmHg, whereas inhibition of the channels enhanced the myogenic response at 20 mmHg (Zhong et al. 2010a). However, there are no studies on the function of Kv7 channels in cerebral arteries from animal models of dementia. Using the Kv7.2‐7.5 activator S‐1 (Bentzen et al. 2006), this study found that Kv7 channel function was attenuated in cerebral arteries of the rTg4510 mice. In order to determine whether the Kv7 channel dysfunction occurred throughout the vasculature, we performed isometric tension measurements on mesenteric arteries from the same mice. In these experiments, S‐1 was equally effective at relaxing mesenteric artery segments from WT and rTg4510 mice. These data suggest that Kv7 channel dysfunction is specific to the cerebral arteries of rTg4510 mice and might contribute to the vascular dysfunction in tauopathies. A functional impairment of Kv7 channels in cerebral arteries is likely to result in increased arterial tone and hypoperfusion of the neuronal tissue, which is characteristic of the vascular dysfunction associated with tauopathies.

Rather than identifying a downregulation in the mRNA encoding for the Kv7 channel *α*‐subunits, particularly KCNQ4 and KCNQ5, we found the ancillary subunit, KCNE5, was downregulated in the rTg4510 mice. KCNE genes encode for single transmembrane‐spanning ancillary subunits, which do not form function channels by themselves, but can regulate the expression, biophysical properties, and pharmacology of certain K^+^ channel *α*‐subunits, particularly the Kv7 channels (McCrossan and Abbott 2004; Kanda and Abbott 2012; Jepps 2017). Previously, KCNE4 and KCNE5 were shown to predominate in rat cerebral arteries, whereas KCNE3 and KCNE4 predominated in rat mesenteric arteries (Jepps et al. 2015; Abbott and Jepps 2016). Thus, given the limited amount of cDNA from the middle cerebral arteries, we focused on these 3 KCNE isoforms and found only KCNE5 to be downregulated. By comparison, in the mesenteric arteries none of the genes investigated showed any differences in expression between WT and rTg4510 mice. Additionally, KCNE5 expression is known to reduce the current density of KCNB1 (which encodes Kv2.1 channels) (David et al. 2015), which plays an important role in cerebral artery reactivity (Zhong et al. 2010b), therefore we also investigated expression of KCNB1 but it was not different in the cerebral arteries between WT and rTg4510 mice.

To obtain some insight into whether a downregulation of KCNE5 in middle cerebral arteries from rTg4510 mice could underlie the impaired Kv7 function in these arteries, we investigated the effect of KCNE5 on the Kv7.4 current. We found Kv7.4 current amplitude and deactivation times were increased in the presence of KCNE5. These data are consistent with the idea that a downregulation of KCNE5 would reduce Kv7 channel function ‐ particularly Kv7.4 channels that are known to be important (Zhong et al. 2010a) ‐ in the cerebral arteries of the tau transgenic mouse.

We also investigated the expression of all KCNQ isoforms, KCNE3‐5 and KCNB1 in the neuronal tissue of the inferior frontotemporal region surrounding the middle cerebral artery of WT and rTg4510 mice. These experiments had two purposes: (1) to investigate if rTg4510 mice have changes in the mRNA expression of these genes, many of which are known to be important in regulating neuronal excitability; and (2) to ensure the expression profiles detected in the middle cerebral arteries were not due to contamination from neurons. In the brain, expression of KCNQ3 and KCNQ5 were downregulated in rTg4510 mice, with the other KCNQ genes unaffected. KCNQ3 and KCNQ5 encode for Kv7.3 and Kv7.5 channels, respectively, which underlie the slowly activating, non‐inactivating M‐current in neurons (Lerche et al. 2000; Shah et al. 2002; Schroeder et al. 2000). Mutations affecting the M‐current are associated with the human epileptic syndrome, benign familial neonatal convulsions (BFNCs) (Cooper and Jan 2003); however this is the first study to show a downregulation of these genes in a tauopathy model. The implications of this finding should be addressed in future studies investigating the impact of NFTs on neuronal function. Additionally, the qPCR analysis found, in contrast to the cerebral arteries, KCNE5 was upregulated in this region of the rTg4510 brain. As mentioned previously, KCNE5 can reduce the current density of KCNB1 (David et al. 2015). Analysis of KCNB1 found that it was downregulated in the brains of rTg4510 mice compared to WT. These data suggest that if KCNE5 subunits were to constitute an interaction partner of Kv2.1 channels in neurons, the upregulation of KCNE5 observed in this study might negatively regulate Kv2.1 channel expression leading to a reduced current density. Future studies should investigate the implications of Kv2.1‐KCNE5 and this mechanism in neurodegenerative diseases associated with NFTs.

We are aware that this study represents a preliminary investigation of Kv7 channel function in cerebral arteries of a tauopathy mouse model, as such there are multiple limitations regarding these findings. First, the wire myography data does not give an indication of how the myogenic response is affected in the cerebral arteries of the rTg4510 mice. It will be important for future studies to perform pressure myography experiments to elucidate how the myogenic response is altered in relation to impaired Kv7 channel function. Second, since the mice were 11 months old, we are unable to determine whether the functional impairment of Kv7 channel responses is a contributory cause or consequence of the hyperphosphorylated tau. It is possible that hyperphosphorylated tau has a direct effect in the cerebral arteries leading to an important contributory cause of the reduced blood flow associated with tauopathies. Future experiments should investigate younger rTg4510 mice to determine when Kv7 channel function becomes impaired relative to the occurrence of NFTs. Third, we were unable to show any direct interaction of KCNE5 and Kv7.4 due to a lack of available tissue and poor KCNE5 antibodies. It is not known if KCNE5 binds to Kv7.4 in cerebral artery myocytes, therefore we do not know if the Kv7 channel impairment is a direct result of KCNE5 downregulation. We hope to be able to resolve this in future studies. Finally, this study only investigated the function of Kv7 channels in these arteries. We believe it is highly probable that other ion channels are affected in the cerebral arteries of the rTg4510 mice. Identifying a functional impairment of Kv7 channels is a starting point that we hope will stimulate future investigations into other ion channels regulating cerebral blood flow in tauopathy models.

Taken together, these data indicate that Kv7 channel function is impaired in cerebral arteries of tau transgenic mice, which may be a contributing factor to the changes in vascular smooth muscle behavior that are associated with increased cerebral artery resistance and decreased cerebral blood flow in tauopathies (Perry et al. 1998; Vidal et al. 2000; Zhao et al. 2014; Bradley et al. 2002; Stopa et al. 2008; Merlini et al. 2016; Qiu et al. 2016). This study has identified a downregulation of the single transmembrane‐spanning ancillary subunit encoded for by KCNE5 in the cerebral arteries of tau transgenic mice, which, given its functional effect on Kv7.4 channels, could represent the mechanism underlying the functional attenuation of Kv7 channels in the cerebral arteries, specifically. It is still unknown whether KCNE5 interacts with Kv7.4 in native smooth muscle cells of the cerebral artery, thus it is also possible that other mechanisms are responsible for the impaired Kv7 channel function in the cerebral arteries of the rTg4510 mice. Nonetheless, impaired Kv7 channel function is likely to lead to increased cerebral artery tone, particularly in the medium‐sized cerebral arteries, thereby increasing pulsations experienced by the smaller, thinner arterioles and capillaries, which increases microvascular damage contributing to the AD‐related microvascular pathology. It is important to note that the vascular pathology associated with AD is multi‐factorial involving degradation of elastin and loss of *α*‐smooth muscle actin (Merlini et al. 2016). Many of these structural abnormalities have been investigated previously; however this study makes important advances into our understanding of how smooth muscle behavior and contractility is affected in AD‐related vascular pathology contributing to the neurone‐hostile environment. Future investigations into such mechanisms may offer improved therapeutic strategies to protect against or retard tauopathies.

## Conflict of Interest

There are no conflicts of interest to report in this study.
